# Chronic pain management for older adults in ambulatory care: Results of a randomized pragmatic trial in Berlin, Germany

**DOI:** 10.1007/s00391-025-02429-1

**Published:** 2025-02-24

**Authors:** Daniela Koios, Arlett Wenzel, Ronny Kuhnert, Christina Rank, Thomas Günther Riemer, Magdalena Glawe, Reinhold Kreutz, Dagmar Dräger

**Affiliations:** 1 Institute of Medical Sociology and Rehabilitation Science, Charité—Universitätsmedizin Berlin, Berlin, Germany; 2 Institute of Clinical Pharmacology and Toxicology, Charité—Universitätsmedizin Berlin, Berlin, Germany

**Keywords:** Chronic pain, Pain therapy, Outpatient care, Aging in place, Nursing, Chronischer Schmerz, Schmerztherapie, Ambulante Versorgung, Pflege

## Abstract

Chronic pain is a major health challenge in older populations and approaches to improve ambulatory care are urgently needed. We conducted a pragmatic trial to test whether staff-directed interventions can improve chronic pain management in older community-dwelling adults and thereby improve their pain situation. Participants of 22 ambulatory nursing services (clusters) were allocated to 3 study arms: I1 (individual intervention with recommendations for each participant’s physician and a newly trained pain nurse), I2 (digital training offered for participants’ physicians and nursing staff) and CG (control group). Survey-based face to face interviews were held at the participants’ homes. Descriptive statistics, ANOVA, and χ^2^-tests were utilized for data analysis. At baseline, 190 and at follow-up 144 participants were analyzed (24% dropout). Overall, the interventions were only implemented by a small proportion of involved staff. We found significant changes in documented nursing care in I1 but improvements regarding pain medication appropriateness or pain situations of participants could not be achieved. Structural challenges like time and staff shortages as well as the lack of billing options in ambulatory care were identified as major obstacles to substantially improve pain care. Policy makers need to enable appropriate compensation models for chronic pain care at home to enable aging in place for a growing population of older adults.

## Abstract (de)

Chronischer Schmerz ist eine große gesundheitliche Herausforderung bei älteren Menschen, und Verbesserungsansätze in der ambulanten Pflege sind dringend erforderlich. Ein pragmatisches Studiendesign sollte testen, ob Komplexinterventionen die Schmerzversorgung und Schmerzsituation älterer Pflegebedürftiger verbessern können. Teilnehmende aus 22 Pflegediensten (Cluster) wurden in drei Studienarme aufgeteilt: I1 (individuelle Intervention mit Empfehlungen für Hausärzt:innen und eine neu ausgebildete Pain-Nurse), I2 (digitale Schulung für Pflegekräfte und Hausärzt:innen) und CG (Kontrollgruppe). Fragebogenbasierte Interviews wurden in der eigenen Häuslichkeit durchgeführt. Deskriptive Statistik, ANOVA und χ^2^-Tests wurden in der Datenanalyse eingesetzt. Zu T0 wurden 190 Teilnehmende analysiert, bei T1 noch 144 (Dropout-Rate: 24 %). Insgesamt wurde die Intervention nur von einem kleinen Teil des beteiligten Personals implementiert. Die Schmerzdokumentation wurde in I1 signifikant verbessert, Verbesserungen in der Angemessenheit der Schmerzmedikation oder der Schmerzsituation der Teilnehmenden konnten jedoch nicht erreicht werden. Strukturelle Herausforderungen wie Zeit- und Personalmangel sowie fehlende Abrechnungsmöglichkeiten für ambulante Schmerzversorgung wurden als wichtige Barrieren identifiziert. Entsprechende Abrechnungsmodelle müssen für ambulante Schmerzversorgung ermöglicht werden, um „aging in place“ in einer alternden Gesellschaft zu ermöglichen.

## Introduction

Chronic pain affects not only those who suffer individually but also health systems in general. The prevalence rises with age [20] and the proportion of older people in countries such as Germany is rapidly increasing. Many of those with a certified need for care are being cared for at home [17] and solutions for chronic pain care in this setting are therefore urgently needed. Overall, older people tend to be excluded from pain trials [12] and, even in geriatric pain research, the ambulatory care sector is often underrepresented (e.g., [13]). To our knowledge, this is the first study exploring interventions to improve chronic pain care for community-dwelling older adults with care needs.

## Research question

A preceding study found a high burden due to chronic pain [4] with substandard pain management in this target group [19]. Hence, the goal for this project was to test whether interventions can positively influence participants’ pain management and thereby reduce pain intensity and related interferences. A relative reduction of severest pain intensity by 20% was defined as the primary outcome. Secondary outcomes were an absolute reduction of severest pain scores by 1.5 points and a decrease in pain interferences. Furthermore, the proportion of participants with an appropriate level of pain medication and pain management nursing care was defined as secondary outcomes and the number of physician contacts was recorded.

## Methods

### Design and setting

This analysis is part of the recently completed “ACHE-Intervention” study, a cluster-randomized trial, conducted in Berlin (Germany), funded by the National Association of Statutory Health Insurance Funds (GKV Spitzenverband). To address the need for real-world evidence [21], we chose a pragmatic trial design. The study was approved by the Charité ethics committee in 02/2022 (EA2/299/21) and conducted in accordance with the Helsinki declaration. Written and informed consent was obtained from all participants, in some cases through their legal guardians. A financial incentive was given to nursing services to compensate for work hours spent on the project.

**Fig. 1:**
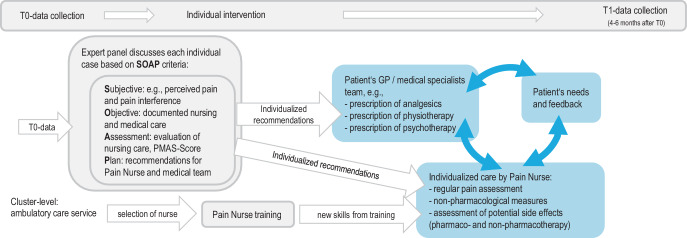
Flowchart showing the planned workflow of study arm I1 (individual intervention)

The study had three arms:

- I1 (Individual intervention, see Fig. 1): one staff member of each ambulatory nursing service was trained as a pain nurse. After T0, each participant’s case was individually discussed in a multidisciplinary (pharmacology, gerontology, medical and nursing science) virtual meeting. Appropriate measures to improve each participant’s pain care were documented. Next, the responsible pain nurse and the participant’s physician were contacted separately with individual recommendations for their patient’s pain management. Each professional should then put the recommendations into practice as appropriate.
- I2 (Digital staff training): in this group, all staff of each participating ambulatory care service as well as the physicians of each participant were offered a digital training regarding chronic pain care for older people.
- CG (Control group): staff of participants in this group were not offered any consulting or training (i.e., standard of care pain management was continued). Participating care services were offered the digital training after data collection.

### Study population

We cooperated with four organizations in the urban area of Berlin and participants were recruited from May 2022 until July 2023. To enable randomization, each care provider had to have at least three suburb-based home nursing services that were willing to participate. Trained study nurses accompanied these employees into the homes of clients as this approach has been identified as most effective in a previous project [16]. Inclusion criteria were age (≥ 65 years), a certified need for care according to the German Long-Term Care Insurance Act, chronic pain (≥ 3 months) and the ability to self-report (min-mental state examination, MMSE ≥ 18). Patients in palliative care were excluded.

### Data collection

Survey-based face to face interviews were conducted by six trained study nurses in the participants’ homes at baseline (T0) and 4–6 months later (T1). Pain characteristics, demographics and information on pain management strategies were collected through self-report. Information on pain-related activities by home nursing providers was obtained by analyzing each participant’s nursing documentation. Information on medication was systematically gathered by scanning packages using barcode scanners and the Instrument for Database-assisted Online recording for Medication (IDOM software) [11]. Additionally, participants who were randomized into the intervention groups had to release their physicians from confidentiality to enable information exchange with the doctors. Participants were blinded to group allocation but, due to the nature of the intervention, providers and interviewers could not be blinded.

### Instruments and measures

Pain intensity and related interferences were assessed with the Brief Pain Inventory (BPI) [14], functional status was evaluated by the Barthel Index (BI) [9], comorbidities with the Age-adjusted Charlson Comorbidity Index (ACCI) [2] and the mini–mental state examination (MMSE) for ability to self-report [5]. Demographic information was collected through structured lists. The pain medication appropriateness scale (PMAS) was utilized to assess the appropriateness of prescribed pain medicine [7], while the appropriateness of pain-related nursing activities was evaluated with an instrument that was developed in the previous cross-sectional study ACHE [19], which is based on the expert standard for pain management nursing care [3].

### Data analysis

We used descriptive statistics for demographics, PMAS, nursing care, and pain items. Distributions of numeric variables were determined by the Kolmogorov-Smirnov test and ANOVA or non-parametric tests were used as appropriate. For categorical data, χ^2^-tests were used to determine differences between groups.

The significance level was set at α = 0.05 with 95% confidence intervals (CI). Data analysis was conducted with IBM SPSS Statistics for Windows, version 27.0 (IBM Corp, Armonk, NY, USA) and R (version 4.4.2). R‑package *srvyr* was used to account for potential cluster effects of nursing services.

## Results

**Fig. 2:**
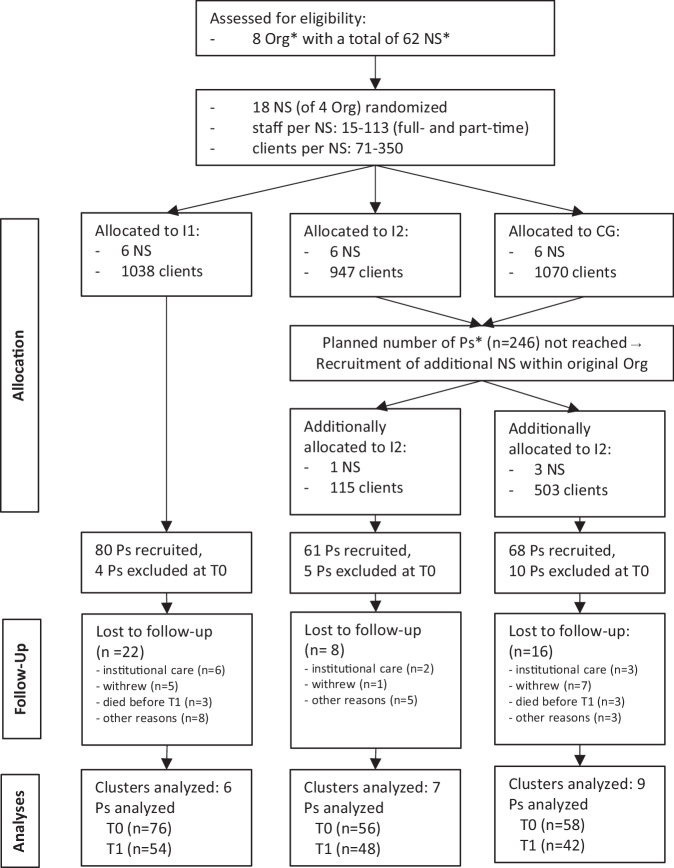
Participant flowchart. *Org* nursing care organization, *NS* nursing care service, *I1* intervention 1, *I2* intervention 2, *CG* control group, *Ps* participants

At baseline, we included 190 older adults with chronic pain (see Fig. 2). The total dropout rate was 24% (*n* = 46), mostly because participants withdrew (*n* = 13), moved into institutional care (*n* = 11), or died (*n* = 6).

The proportion of women was higher than men but did not differ significantly between study arms (see Table 1). Similarly, most demographic characteristics were distributed evenly, except for education and comorbidity levels.

**Table 1:** Baseline characteristics

	I1	I2	CG	Total	*p*-value*
Number of participants	76 (40%)	56 (29.5%)	58 (30.5%)	190 (100%)	–
*Sex*					
Female	59 (77.6%)	40 (71.4%)	45 (77.6%)	144 (75.8%)	0.663
Male	17 (22.4%)	16 (28.6%)	13 (22.4%)	46 (24.2%)	
*Age*					
Age in years M (SD)	85.3 (7.1)	84.4 (6.9)	83.4 (7.2)	84.5 (7)	0.316
Range years	(67–101)	(67–94)	(65–98)	65–101	–
*Age groups*					
65–74 years	6 (7.9%)	6 (10.7%)	8 (13.8%)	20 (10.5%)	0.499
75–84 years	25 (32.9%)	18 (32.1%)	24 (41.4%)	67 (35.3%)	
≥ 85 years	45 (59.2%)	32 (57.1%)	26 (44.8%)	103 (54.2%)	
*Educational level (ISCED)*					
Low	17 (22.4%)	5 (8.9%)	4 (6.9%)	26 (13.7%)	< 0.001
Medium	47 (61.8%)	44 (78.6%)	33 (56.9%)	124 (65.3%)	
High	12 (15.8%)	7 (12.5%)	21 (36.2%)	40 (21.1%)	
*MMSE*					
Sum score M (SD)	26.8 (2.7)	26.5 (3.1)	26.2 (3.1)	26.6 (2.9)	0.493
Range	(19–30)	(18–30)	(18–30)	18–30	–
*Barthel Index*					
Sum score M (SD)	77.6 (18.2)	79.6 (14.4)	80.3 (17.4)	79 (16.9)	0.63
Range	(5–100)	(35–100)	(15–100)	5–100	–
*Care grade (impairment)*					
1 (slight)	5 (6.6%)	3 (5.4%)	8 (13.8%)	16 (8.4%)	0.465
2 (considerable)	41 (53.9%)	30 (53.6%)	26 (44.8%)	97 (91.1%)	
3 (severe)	30 (39.5%)	23 (41.1%)	24 (41.4%)	77 (40.5%)	
*CCI*					
Low	–	–	**–**	–	0.03
Moderate	7 (9.2%)	13 (23.2%)	5 (8.6%)	25 (13.2%)	
Severe	69 (90.8%)	43 (76.8%)	53 (91.4%)	165 (86.8%)	

* *p*-value as result of χ^2^-test for categorial variables and ANOVA for numerical variables

*M* mean, *SD* standard deviation, *CCI* Charlson Comorbidity Index, *ISCED* International Standard Classification of Education, *MMSE* Mini-mental status examination

### Implementation

During the implementation phase of the project, it became clear that the intervention was not implemented as planned (despite intense efforts of the study team to support relevant actors): Most I1 participants did not receive the individualized intervention as planned, e.g., 31.6% of physicians in I1 (*n* = 76) could not be contacted or generally declined participation. A qualitative assessment of the documented contacts with our participants’ physicians showed that a lack of time was the most common reason for not participating. Of those who were able to take the time to personally talk about our intervention with our study physician (*n* = 39), the recommended changes were positively perceived by only 46.2%. The other physicians did not agree (35.9%) or only partially (17.9%) agreed with the recommendations from our study team. Some reasons for declining recommendations were that the patients had rejected such treatment in the past (e.g., certain medications) or a lack of motivation for recommended measures (e.g., exercise). In addition to the limited implementation on the side of the physicians, only three pain nurses (out of six) visited the participants as often as planned. To illuminate the barriers in this context, an additional qualitative analysis was conducted and showed that a perceived lack of knowledge acquisition and equipment led to some reluctance to take on responsibilities, hindering implementation [15]. Similarly, the digital training (I2) was only completed by one third of nursing staff who enrolled. A voluntary evaluation completed by 17 nurses who did enrol but did not complete the training, showed that a lack of time and technical problems were the main reasons for not participating. Physicians did not participate at all in the offered online training.

### Effects on pain management

**Fig. 3:**
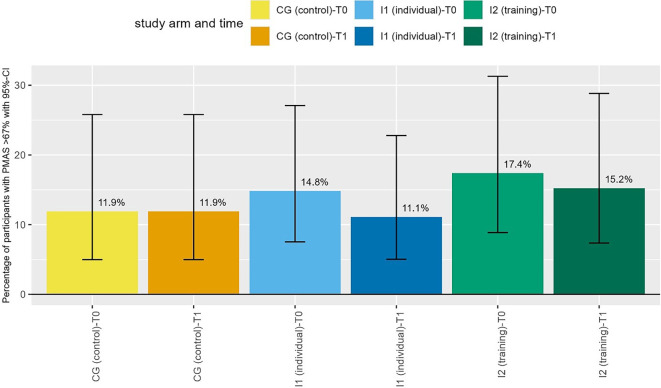
Proportion of participants with appropriate pain medication (PMAS > 67%)

At baseline 12.6% of all participants (*n* = 190) had a PMAS of > 67% (i.e., appropriate pain medication) with proportions varying slightly between study arms (CG: 8.6%, I1: 13.2%, I2: 16.1%). Comparing only those with values for T0 and T1 (total: *n* = 142; CG: *n* = 42, I1: *n* = 54, I2: *n* = 46), our analysis shows that the proportion of those with appropriate pain medication decreased in our intervention groups and remained stable in CG (see Fig. 3). As all 95% CIs overlap when comparing T0 to T1, it seems that there are no statistically significant differences between groups or over time.

**Fig. 4:**
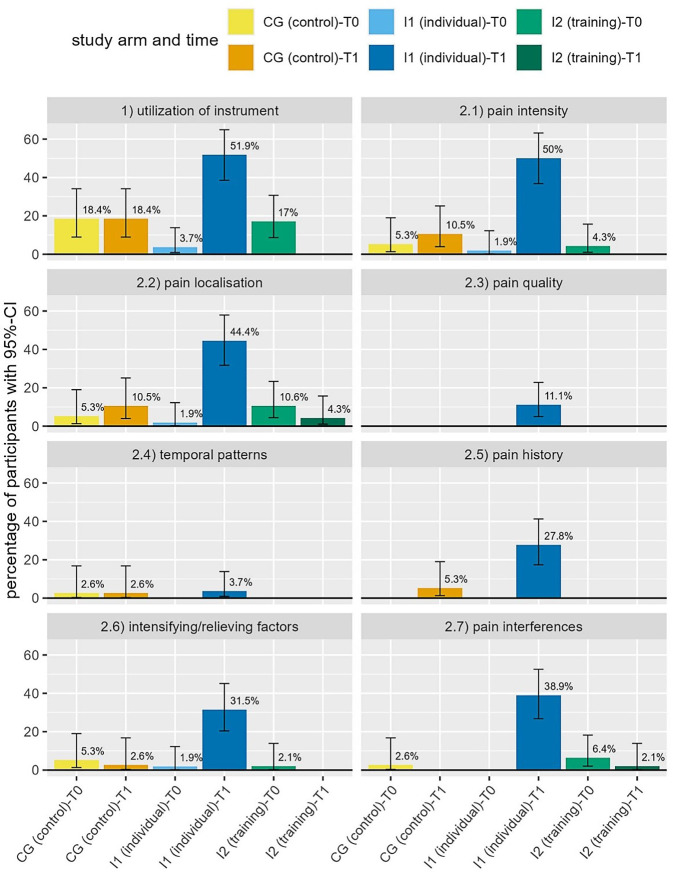
Proportion of participants regarding pain assessment

According to the expert standard for pain management nursing care [3], a systematic instrument should be regularly used to assess patients’ pain situation. At baseline, such an instrument was only documented for 10.8% (*n* = 185). These regular pain assessments should include seven items but their overall utilization seems low: At baseline, only 4.9% of all available nursing documentation included information on pain localization and 3.2% documented pain intensity. Pain interferences were documented for 2.7%, intensifying and relieving factors for 2.1% of the participants and only 2 (1.1%) documentations included information on when and for how long pain occurs. None documented information on pain quality or history. Figure 4 compares these measures only for those with values at T0 and T1 (total: *n* = 139; CG: *n* = 38, I1: *n* = 54, I2: *n* = 47), showing the proportion of participants in each group for which each item is documented with the 95% CI for each item. In I1, there are significant increases of instrument utilization and most documented assessment items as the 95% CIs do not overlap when comparing T0 to T1 in this group.

Appropriate nursing pain management should also entail an individual care plan including education and nonpharmacological interventions. For the latter, effects (desired and undesired) should be monitored and documented. Similarly, pain medication effects should be monitored. No participant’s nursing documentation included all of these measures at any point, meaning that none fulfilled the requirements of the expert standard [3]. Consequently, the proportion of those with appropriate nursing pain care could not be assessed despite considerable improvements in documented nursing pain care in I1.

Additionally, participants were asked how often they had seen their physician within the last 3 months. At baseline, the majority (53.2%, *n* = 187) saw their physician once within this period, followed by two (15.3%), none (13.7%) and three (12.1%) physician contacts. The mean difference of physician contacts (i.e., difference between contacts at T0 and T1 for each participant) was assessed per study group and shows a minimal reduction of contacts in I1 (−0.16, 95% CI −0.35–0.03) and minimal increases in I2 (0.08, 95% CI −0.41–0.58) and CG (0.12, 95% CI −0.35–0.59). As all 95% CIs overlap and contain 0, there are no significant differences.

### Effects on participants’ pain situation

The most common pain-related diagnoses in our sample were low back pain (82.1%), osteoarthritis (75.8%) and neuralgia (55.3%). At baseline, severest pain showed a mean of 6.3 (out of 10 for BPI item 3) points and 32.9 points (out of 70 for sum of BPI items 9A–9G) for pain interferences. Comparing only those participants with values for T0 and T1, we found differences between study arms regarding severest pain at baseline, being lower in I1 (see Table 2).

**Table 2:** Pain measures

Severest pain	Total (*n* = 138)	CG (*n* = 40)	I1 (*n* = 50)	I2 (*n* = 48)	*p*-value*
T0: M (95% CI)	6.2 (5.7–6.6)	6.3 (5.9–6.7)	5.6 (5.1–6.1)	6.7 (5.9–7.5)	0.047
T1: M (95% CI)	6.2 (5.8–6.6)	6.5 (5.9–7)	5.8 (5.4–6.3)	6.4 (5.6–7.3)	0.447
*Interference sum score*	*Total (n* *=* *112)*	*CG (n* *=* *30)*	*I1 (n* *=* *41)*	*I2 (n* *=* *41)*	*–*
T0: M (95% CI)	30.5 (27.1–33.8)	33.3 (29.9–36.7)	30.8 (25.2–36.4)	28.1 (21.4–34.7)	0.413
T1: M (95% CI)	31.8 (28.6–35)	32.7 (27.3–38)	32.2 (28.6–35.7)	30.8 (23.9–37.7)	0.88

* *p*-value for comparison between study arms

*M* mean, *CI* confidence interval

Severest pain in the individual intervention group increased by 3.6% and by 3.2% in the control group. Only participants in the digital training group indicated a reduction in pain intensity by 4.5%. These effects were not statistically significant in any group. Hence, the goal to reduce severest pain intensity by 20% (primary outcome) or by 1.5 points (secondary outcome) was not achieved. Similarly, the sum score of pain-related interferences was not significantly reduced in any of the groups. The mean score increased by 4.5% for I1 participants and by 9.6% in I2, while CG participants showed a reduction of pain related interferences by 1.8%. Additionally, we assessed the relative and absolute difference of pain intensity and pain interference sum score on a participant level, which also showed no relevant effects.

## Discussion

Overall, neither the individual intervention nor the staff training could improve older community-dwelling adults’ chronic pain situation in this pragmatic trial design. Comorbidity levels show a high burden with the vast majority scoring more than 5 CCI points which is also reflected in high proportions of considerable and severe impairment (i.e., care grades). Targeting this highly vulnerable group in research is necessary [13] but it comes with the risk that multimorbidity can complicate treatment and the measurement of effects. While none of the pain-related effects were significant, the slight increase of pain interferences might be rooted in a more intense focus on pain in our participants’ daily life. This may have been caused by the participation in the study itself or (for I1) by increased frequency of pain assessments by nursing staff. As the planned interventions were only implemented for few participants, it is not possible to estimate whether broader implementation would have produced different results.

Compared to a previous cross-sectional study [19] our baseline data shows a severe decline in pain-related documentation items, e.g., pain location was then documented for 38.5% and now only for 4.9%. Overall, results need to be interpreted cautiously due to sample size and as there are multiple items with no documentation (e.g., pain quality for most groups). While documented pain management activities of nursing staff increased in I1, it has not reached the level recommended by the expert standard [3]. Reasons for not reaching this level can be broadly attributed to three aspects [15]: firstly, a shortage of nursing staff impeded implementation of our intervention. Secondly, the pain nurses’ training was not tailored to the ambulatory care sector and left participants feeling that they still lack knowledge to take on responsibilities. Lastly, a lack of standardized tools, documentation possibilities, and opportunities to initiate multidisciplinary cooperation (e.g., with physicians) hindered a holistic pain management strategy.

Furthermore, the proportion of those with appropriate pain medication [7] could not be increased. Severe deficits in pain medication in this population was reported previously [16] but with 12.6% at baseline, the proportion of those with a PMAS of > 67% was even smaller in our current study (compared to 18%). These results emphasize the urgent need for interventions targeted at physicians who care for older people; however, our study also showed that it is difficult to involve general practitioners due to widespread time scarcity.

Our study did not achieve the desired effects but it remains unclear whether the interventions could have improved older peoples’ chronic pain situation if it had been implemented as planned. The following section therefore discusses potential reasons for failing to implement the intervention as well as recommendations. These aspects can be categorized in three areas:

1. Project-related: contrary to our expectations, the training as pain nurse (I1) did not enable the nurses to confidently implement changes in pain care in a self-directed manner. Based on the results of the additional qualitative analysis [15], we recommend that targeted staff are involved early in the planning process for future projects to enable tailored training contents which might lead to greater commitment, also regarding the participation in online training (I2). For physicians, nonparticipation was potentially rooted in the fact that we could not offer continuing education credits due to the interventional nature of our study (as these can only be awarded for training that are open to all physicians in Germany). While this was not explicitly expressed by any of the contacted physicians, experience from previous projects suggests that participation could be higher when such credits can be offered. For nursing staff members the evaluation showed that the digital format caused difficulties for many. Consequently, face to face training might have been more suited in this target group and should be considered in future projects.
2. Setting-related: a lack of time and workforce has negatively impacted both interventions. For I1, staff shortages and the generally high load of tasks in this setting have substantially hindered implementation by nursing staff. Financial incentives were paid in this group but this cannot create resources that are simply not there. Hence, structural changes are required in this sector. While the urgent need for more nursing staff in Germany is hardly new knowledge (e.g., [8]) the results of this project support these findings especially for the ambulatory home care sector. To enable aging in place for a rapidly growing older population, the strengthening of multidisciplinary approaches within ambulatory care services is urgently needed and was also identified as important topic by the pain-nurses of our project [15]. Similar to nursing staff, many primary care physicians have also cited a lack of time for not being able to participate in our study (both in I1 and I2). This probably impeded changes in prescribed medication and nonpharmacological treatment. We suggest that further setting-specific studies explore how strengthening of specialized ambulatory nursing care may relieve pressure from physicians and other primary care providers, which could ultimately result in both better care and reduced costs [10]. Additionally, specialized nursing with more responsibilities can increase job attractiveness, addressing the lack of skilled staff in this sector.
3. Policy-related: a major challenge lies in the fact that chronic pain care is not billable in ambulatory nursing care [15]. Our project tried to tackle this problem by a small compensation payment for participating nursing services but this was obviously not sufficient. Based on the results of this project and our experiences from previous studies, we think that compensation models for chronic pain care need to be established in this sector, as it cannot be expected of nursing staff to provide chronic pain care without any compensation allocated for it. Based on many years of research in the ambulatory setting (e.g., [4, 19]) a compensation model similar to palliative care appears feasible from our viewpoint as pain management is also an important part of these services [6]. The German healthcare system is considered one of the most expensive systems globally [1] and public funds are already stretched. Consequently, covering additional services is not easily argued for; however, multidisciplinary pain rehabilitation programs are considered more cost-effective than invasive measures [18], and we believe that an investment in ambulatory pain care could therefore prove to be less burdensome for public funds in the long run. Hence, efforts to establish compensation for ambulatory nursing pain care could not only take some pressure off physicians and other health professionals but it could eventually turn out to be more cost-effective.

## Limitations

Firstly, we did not reach our planned sample size and, secondly, the interventions were not implemented as designed. It is therefore not possible to determine whether the planned interventions could have improved older adults‘ pain situation under different circumstances. Additionally, our sample consisted of older adults (≥ 65 years) living in the city of Berlin, Germany, mostly of European descent, meaning that results are not generalizable to older adults living in more rural areas or to populations differing in ethnicity or age.

## Practical conclusion

While our intervention (directed at medical and nursing staff) was mostly not implemented as planned, it has partially improved pain management practices; however, the primary goal to reduce pain intensity or related interferences of participants was not achieved. Our additional qualitative analyses suggest that other training formats might be more suited in the ambulatory setting. For future studies, researchers should consider whether early participatory involvement of targeted staff can be realized as this might increase staff commitment. Based on our experience from other projects in this sector, we assume that structural problems (such as a scarcity of staff and the lack of specific billing options in ambulatory care) hinder efficient chronic pain care. To enable aging in place for a growing population of older adults, policy makers need to urgently enable appropriate compensation models for chronic pain care.

## Data Availability

The data that support the findings of this study are not openly available due to reasons of sensitivity and are available from the corresponding author upon reasonable request. Data are located in controlled access data storage at Charité—Universitätsmedizin Berlin.

## Declarations

Ethical approval was obtained from the Ethics Committee of Charité—Universitätsmedizin Berlin (EA2/299/21), all participants (or their legally authorized representatives) gave written informed consent and the study was conducted in accordance with the Helsinki declaration.
